# Evaluation of high-resolution microarray platforms for genomic profiling of bone tumours

**DOI:** 10.1186/1756-0500-3-223

**Published:** 2010-08-08

**Authors:** Stine H Kresse, Karoly Szuhai, Ana H Barragan-Polania, Halfdan Rydbeck, Anne-Marie Cleton-Jansen, Ola Myklebost, Leonardo A Meza-Zepeda

**Affiliations:** 1 Department of Tumour Biology, The Norwegian Radium Hospital, Oslo University Hospital, Oslo, Norway; 2 Department of Molecular Cell Biology, Leiden University Medical Center, Leiden, The Netherlands; 3 Norwegian Microarray Consortium, Department of Molecular Biosciences, University of Oslo, Oslo, Norway; 4 Department of Informatics, University of Oslo, Oslo, Norway; 5 Department of Pathology, Leiden University Medical Center, Leiden, The Netherlands

## Abstract

**Background:**

Several high-density oligonucleotide microarray platforms are available for genome-wide single nucleotide polymorphism (SNP) detection and microarray-based comparative genomic hybridisation (array CGH), which may be used to detect copy number aberrations in human tumours. As part of the EuroBoNeT network of excellence for research on bone tumours (eurobonet.eu), we have evaluated four different commercial high-resolution microarray platforms in order to identify the most appropriate technology for mapping DNA copy number aberrations in such tumours.

**Findings:**

DNA from two different cytogenetically well-characterized bone sarcoma cell lines, representing a simple and a complex karyotype, respectively, was tested in duplicate on four high-resolution microarray platforms; Affymetrix Genome-Wide Human SNP Array 6.0, Agilent Human Genome CGH 244A, Illumina HumanExon510s-duo and Nimblegen HG18 CGH 385 k WG tiling v1.0. The data was analysed using the platform-specific analysis software, as well as a platform-independent analysis algorithm. DNA copy number was measured at six specific chromosomes or chromosomal regions, and compared with the expected ratio based on available cytogenetic information. All platforms performed well in terms of reproducibility and were able to delimit and score small amplifications and deletions at similar resolution, but Agilent microarrays showed better linearity and dynamic range. The platform-specific analysis software provided with each platform identified in general correct copy numbers, whereas using a platform-independent analysis algorithm, correct copy numbers were determined mainly for Agilent and Affymetrix microarrays.

**Conclusions:**

All platforms performed reasonably well, but Agilent microarrays showed better dynamic range, and like Affymetrix microarrays performed well with the platform-independent analysis software, implying more robust data. Bone tumours like osteosarcomas are heterogeneous tumours with complex karyotypes that may be difficult to interpret, and it is of importance to be able to well separate the copy number levels and detect copy number changes in subpopulations. Taking all this into consideration, the Agilent and Affymetrix microarray platforms were found to be a better choice for mapping DNA copy numbers in bone tumours, the latter having the advantage of also providing heterozygosity information.

## Background

Chromosomal aberrations are frequent in cancer, and change in gene dosage is a common mechanism for activation or attenuation of oncogenes and tumour suppressor genes, respectively. In order to precisely identify chromosomal regions of gain and loss, a number of microarray-based technologies have been developed to measure genome-wide DNA copy number [1-3]. Microarray-based comparative genomic hybridisation (array CGH) provides the means of quantitatively measuring DNA copy number aberrations at high-resolution and map them directly to the genome sequence.

High-density oligonucleotide microarrays contain synthetic single-stranded oligonucleotide probes, and different designs for array CGH are offered by a number of companies. The size of the oligonucleotides ranges from 25-mer to 85-mer depending on the type of microarray. Some of these microarrays have been developed for linkage analysis by the identification of single nucleotide polymorphisms (SNPs), allowing the simultaneous detection of DNA copy number changes and loss of heterozygosity (LOH), which is the regional loss of the contribution to the genome from one parent [4,5]. LOH can be copy number neutral when the deleted chromosomal region is compensated by mitotic recombination, resulting in homozygosity without physical DNA loss.

With the increasing number of microarray formats available for detection of DNA copy number changes and LOH, with differences in design, resolution and experimental information obtainable, there is a need to evaluate the alternatives in order to identify the best microarray platform for specific studies. An increasing number of comparative studies of high-resolution platforms have been performed [6-15], addressing different types of research questions. In general, most platforms have been reported to perform well, but differences occur, and obviously each platform has its advantages and disadvantages that need to be taken into consideration.

Benign and malignant bone tumours are often found in bone and show varying degrees of osteogenic, chondrogenic, fibrogenic or neuroectodermal differentiation, among others. Primary malignant bone tumours, or bone sarcomas, arise frequently in the long bones of the extremities [16]. Most tumours arise sporadically, but some occur in relation to pre-existing conditions of bone or inherited diseases. Bone sarcomas can occur at any age, but the tumours frequently occur in children and adolescents in addition to older people. About 35% of the bone sarcomas are osteosarcomas, 25% are chondrosarcomas and 16% are Ewing sarcomas/PNET (Primitive NeuroEctodermal Tumour) [16]. Most conventional osteosarcomas have complex karyotypes with numerous numerical and structural aberrations, whereas the karyotype complexity is in general reduced from chondrosarcomas to Ewing sarcoma/PNET [16]. Both high level of amplification and homozygous deletion of various sizes can occur in these tumour types. Due to their complex karyotype, microarray-based technologies are particularly useful to decipher chromosome aberrations in these tumours.

As part of the EuroBoNeT European network of excellence for research on bone tumours (European Network to Promote Research into Uncommon Cancers in Adults and Children: Pathology, Biology and Genetics of Bone Tumours, eurobonet.eu), we have evaluated four different high-resolution commercial microarray platforms for DNA copy number analysis; Affymetrix Genome-Wide Human SNP Array 6.0, Agilent Human Genome CGH 244A, Illumina HumanExon510s-duo and Nimblegen HG18 CGH 385 k WG tiling v1.0, in order to identify the most appropriate microarray platform for mapping DNA copy number aberrations in bone tumours.

## Materials and methods

### Test material

Two different cell lines were selected for the testing; TC-32, an Ewing sarcoma/PNET cell line with simple chromosomal aberrations, and OSA (termed SJSA-1 by ATCC), a conventional osteosarcoma cell line with complex genomic rearrangements. The TC-32 cell line was kindly provided by Dr. S.A. Burchill (St. James' University Hospital, Leeds, UK), and the SJSA-1 (OSA) cell line was obtained from ATCC.

TC-32 was cultured in RPMI 1640 GlutaMAX medium supplemented with 20% foetal calf serum and penicillin/streptomycin (all Invitrogen, Carlsbad, CA, USA). OSA was grown in RPMI1640 (Lonza, Basel, Switzerland) supplemented with 10% foetal calf serum (PAA Laboratories GmbH, Pasching, Austria), GlutaMAX (Invitrogen) and penicillin/streptomycin (Lonza), at 37°C with 5% CO_2_. All cells were split when reaching 80% confluency.

DNA was isolated from each cell line using the High Pure PCR Template Isolation Kit (Roche, Basel, Switzerland) following the manufacturer's instructions. For two channel arrays (Agilent and Nimblegen), a commercial pooled male DNA was used as a reference (Promega, Madison, WI, USA), while for single channel arrays (Affymetrix and Illumina), data from a normal sample set provided by each supplier was used as a normal reference control. The same DNA preparations from the cell lines, reference DNA and Human Cot-1 DNA (Invitrogen) were used for all platforms and replicates in order to avoid effects of DNA quality or cell line variability on the results.

### Microarray platforms

The cell lines were profiled for DNA copy number changes in duplicate using four different high-resolution oligonucleotide microarray platforms; Affymetrix Genome-Wide Human SNP Array 6.0, Agilent Human Genome CGH 244A, Illumina HumanExon510s-duo and Nimblegen HG18 CGH 385 k WG Tiling v1.0. Information and characteristics for each platform are summarized in Table 1. Hybridisations for Affymetrix and Nimblegen microarrays were carried out at the Microarray Core Facility at The Norwegian Radium Hospital (Oslo, Norway), whereas hybridisations for Agilent and Illumina microarrays were performed at Service XS (Leiden, The Netherlands). For all platforms, the suppliers' protocols were strictly followed. All datasets can be viewed in the microarray depository ArrayExpress (http://www.ebi.ac.uk/microarray-as/ae/, accession number E-TABM-805).

**Table 1:** Microarray platform characteristics

Platform	Technology	Sample labelling	Sample requirement	Number of features	Median probe spacing	Analysis
**Affymetrix Genome-Wide Human SNP Array 6.0**	Oligonucleotide (25 nt)	PCR reduction	0.5 μg	1,852 k	< 700 bp	DNA copy number and LOH

**Agilent Human Genome CGH 244A**	Oligonucleotide (60 nt)	Whole genome	0.5-1 μg	236 k	8,900 bp	DNA copy number

**Illumina HumanExon510s-duo**	Oligonucleotide (25 nt)	PCR reduction	0.75 μg	511 k	3,200 bp	DNA copy number and LOH

**Nimblegen HG18 CGH 385 k WG Tiling v1.0**	Oligonucleotide (50-75 nt)	Whole genome	1 μg	385 k	6,270 bp	DNA copy number

Characteristics of the different high-resolution microarray platforms evaluated.

#### Affymetrix Genome-Wide Human SNP Array 6.0

The Affymetrix Genome-Wide Human SNP Array 6.0 contains more than 1.8 million genetic markers, including more than 906,000 SNP probes and more than 946,000 probes designed for detection of DNA copy number changes. Quality control and normalization were performed in Genotyping Console v3.0.1 (Affymetrix). Data was quality controlled using the contrast quality control (CQC) algorithm with a minimal call rate of > 86%. DNA copy number analysis was performed in Genotyping Console using quantile probe level normalization, regional GC correction and default settings. Segmentation was performed using an HMM algorithm, and segments were reported as copy number states (i.e. 0, 1, 2, 3 and ≥4 copies).

#### Agilent Human Genome CGH 244A

The Agilent Human Genome CGH 244A microarray contains approximately 236,000 *in situ *synthesized 60-mer oligonucleotides spanning coding and non-coding regions. Microarray images were processed in Agilent Feature Extraction v9.1 using default settings (no background subtraction and spatial detrend), as well as "ranked consistent probe methods" for normalization. Export files were further analysed using Agilent DNA Analytics v4.0.76. Aberrations were detected using the AMD-2 algorithm with a threshold of 8.0, and a filter against aberrations with less than six probes was used.

#### Illumina HumanExon510s-duo

The HumanExon510s-duo microarray is a gene centric array, having the majority of the 511,354 contained markers within or near genes. More than 60% of the markers are located within 10 kb of a gene, and the probes provide coverage for 99.9% of the RefSeq genes. In addition, probes for known regions of copy number variation are included. Fluorescent intensities from the scanner were imported into BeadStudio v3.3.4 (Illumina) and normalized using quantile normalization. DNA copy number analysis was performed using the cnvPartition algorithm v1.2.0 and default settings.

#### Nimblegen HG18 CGH 385 k WG Tiling v1.0

The Nimblegen HG18 CGH 385 k WG Tiling v1.0 microarray contains 385,000 long oligonucleotides (50-75-mer) that tile the human genome. Microarray images were processed in NimbleScan v2.3 (Nimblegen), and segmentation was performed using the DNACopy algorithm with default settings.

#### Platform-independent analysis

Platform-independent analysis was performed in Nexus (BioDiscovery, El Segundo, CA, USA) using the rank segmentation algorithm with default settings (threshold of 0.6 for high gain, 0.2 for gain, -0.2 for loss and -1.0 for big loss). SNP analysis of the Affymetrix and Illumina data was performed in Nexus using the SNP-FASST segmentation algorithm with default settings.

## Results and discussion

Four different commercial high-resolution oligonucleotide microarray platforms, Affymetrix Genome-Wide Human SNP Array 6.0, Agilent Human Genome CGH 244A, Illumina HumanExon510s-duo and Nimblegen HG18 CGH 385 k WG tiling v1.0, were evaluated for genomic profiling of bone tumours. Information and characteristics for each platform are summarized in Table 1. Two different bone sarcoma cell lines, TC-32 (Ewing sarcoma/PNET) and OSA (conventional osteosarcoma), having simple and extensive chromosomal aberrations, respectively, were selected for the testing. Multicolour COBRA-FISH-based karyotyping of the TC-32 cell line has been described earlier [17,18], with the resulting karyotype 48, XX,+i(1)(q10),+8, t(11;22)(q24;q12).

DNA from the cell lines was labelled and hybridised in duplicate to all four microarray types following the manufacturers' instructions, and the same DNA preparations were used for all platforms and replicates in order to avoid effects of DNA quality or cell line variability on the results. Data processing and analysis were performed using the corresponding software for each of the platforms with default settings.

### Reproducibility

To measure the reproducibility of log_2 _ratios between replicate hybridisations, Pearson's correlation was calculated based on all data points (Table 2). Illumina microarrays showed the highest degree of correlation between replicates, 0.96 and 0.94 for TC-32 and OSA, respectively. Agilent microarrays showed also a high correlation between replicates, 0.91 for both cell lines, while Affymetrix microarrays showed an intermediate correlation of 0.75 and 0.84 for TC-32 and OSA, respectively. Nimblegen microarrays showed the lowest correlation, 0.73 and 0.63 for OSA and TC-32, respectively. Although having a more complex karyotype, the OSA cell line showed slightly better correlation for the replicates than the TC-32 cell line for Affymetrix and Nimblegen microarrays. Scatter plots of log_2 _ratios for the replicate hybridisations for all microarray platforms are shown in Additional file 1.

**Table 2:** Reproducibility of replicate hybridisations and signal response to copy number

Platform	Corr TC-32	Corr OSA	0n: *CDKN2A* TC-32		1n: 9p21.3-p21.2 TC-32 **Log**_**2**_**ratio (SD)**	2n: Chr 2 TC-32 **Log**_**2**_**ratio (SD)**	3n: Chr 8 TC-32 **Log**_**2**_**ratio (SD)**	4n: 1q TC-32 **Log**_**2**_**ratio (SD)**	>10n: *MDM2 * OSA	
								
			**Log**_**2**_**ratio (SD)**	Size [kb]					**Log**_**2**_**ratio (SD)**	Size [kb]
**Theoretical value**	1.0	1.0	< -3	-	-1.0	0.0	0.58	1.0	> 3	-

**Affymetrix Genome-Wide Human SNP Array 6.0**	0.75	0.84	-1.79 (0.93)	166	-0.53 (0.43)	0.00 (0.41)	0.34 (0.44)	0.56 (0.44)	1.86 (0.49)	146
			-2.09 (0.92)	148	-0.62 (0.35)	0.00 (0.32)	0.37 (0.34)	0.59 (0.34)	1.99 (0.50)	146

**Agilent Human Genome CGH 244A**	0.91	0.91	-4.72 (1.57)	135	-0.96 (0.31)	0.00 (0.19)	0.52 (0.27)	0.92 (0.22)	4.69 (0.24)	147
			-4.83 (1.53)	135	-0.97 (0.30)	0.01 (0.21)	0.54 (0.29)	0.95 (0.25)	4.74 (0.21)	147

**Illumina HumanExon510 s-duo**	0.96	0.94	-4.72 (1.07)	171	-0.67 (0.54)	-0.05 (0.20)	0.22 (0.29)	0.36 (0.26)	1.24 (0.59)	122
			-4.69 (0.91)	171	-0.67 (0.59)	-0.05 (0.20)	0.22 (0.29)	0.36 (0.26)	1.23 (0.58)	122

**Nimblegen HG18 CGH 385 k WG Tiling v1.0**	0.63	0.73	-0.85 (0.57)	169	-0.45 (0.30)	-0.06 (0.19)	0.24 (0.21)	0.48 (0.24)	2.58 (0.98)	156
			-0.88 (0.50)	169	-0.47 (0.29)	-0.06 (0.20)	0.26 (0.21)	0.53 (0.23)	2.46 (1.0)	156

Pearson's correlation of log_2 _ratios for the replicate hybridisations based on all data points and the average log_2 _ratio with standard deviation for six specific chromosomes or chromosomal regions, representing 0, 1, 2, 3 and 4 copies in TC-32, as well as high-level amplification in OSA, for each hybridisation. In addition, the measured size of the regions for homozygous deletion of the *CDKN2A *locus in TC-32 and high-level amplification of the *MDM2 *locus in OSA is given for each hybridisation.

In a previous study of melanoma cell lines using lower resolution oligonucleotide microarrays from Affymetrix, Agilent and Nimblegen, similar results were observed [8]. Here, the highest resolution array from Agilent tested (185 K) showed the highest correlations (ranging 0.72-0.86 for the different samples hybridised), whereas the highest resolution array from Affymetrix tested (500 K) showed intermediate correlations (0.54-0.67). Also here the Nimblegen array (1500 K) showed the lowest correlations (0.27-0.57). In addition, a significant higher degree of correlation was observed for the higher-density microarrays compared to the lower-density microarrays from the same supplier (Agilent 185 K vs 44 K and Affymetrix 500 K vs 100 K) [8]. Although not directly comparable, this is in concordance with the even higher correlations between replicate hybridisations observed with the higher-resolution microarrays from Agilent and Affymetrix used here.

Similar results have also been observed between replicate hybridisations of Affymetrix 500 K arrays, Agilent 44B arrays and Illumina Hap550 arrays for one leukaemia cell line [14]. The standard deviation for each probe across four replicate hybridisations was calculated, and Illumina arrays showed the lowest median standard deviation (0.059), followed by Agilent (0.083) and Affymetrix (0.101). However, when the median standard deviation was normalized to the number of measurements on each platform, all platforms showed similar levels of variation [14].

### Signal response to copy number

**Figure 1:**
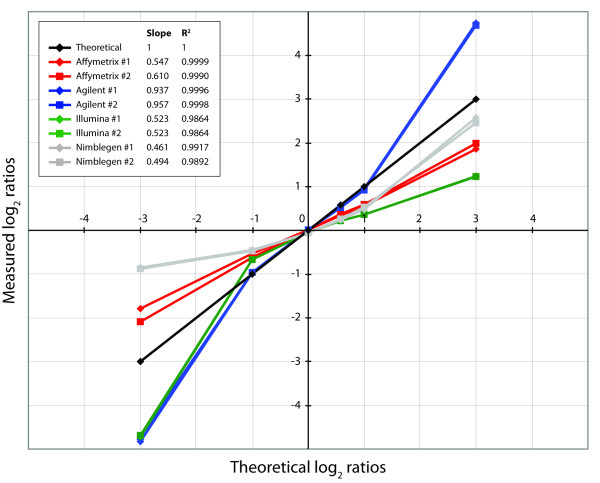
**Plot of measured log_2 _ratios compared to theoretical values for specific chromosomes or chromosomal regions**. Plot of average measured log_2 _ratios compared to theoretical values for six specific chromosomes or chromosomal regions; the *CDKN2A *locus in 9p21.3, 9p21.3-p21.2, chromosome 2, chromosome 8, 1q (all in TC-32) and the *MDM2 *locus in 12q15 (in OSA) (representing 0, 1, 2, 3, 4 and >10 copies, respectively) for all microarray platforms and replicates. The slope and regression coefficient (R^2^) for the regression line based on four of the regions, representing 1, 2, 3 and 4 copies, are given.

To quantify and evaluate the signal response to copy number for each platform, the measured DNA copy numbers for five specific chromosomes or chromosomal regions in TC-32 (representing 0, 1, 2, 3 and 4 copies) and one specific chromosomal region in OSA (representing high-level amplification) were compared with the expected ratios based on available cytogenetic and molecular information. Homozygous deletion of the locus around the tumour suppressor gene *CDKN2A *in 9p21.3, heterozygous deletion of 9p21.3-p21.2, normal copy number of chromosome 2, three copies of chromosome 8 and four copies of the long arm of chromosome 1 were measured in TC-32, and high-level amplification of the locus around the oncogene *MDM2 *in 12q15 was measured in OSA. For both replicate hybridisations, the average log_2 _ratio was determined for each normalized segment, as well as the standard deviation of the signals, and this is presented in Table 2. Figure 1 shows a plot of the measured average log_2 _ratios compared to the theoretical values. In addition, the size of the homozygous deletion of the *CDKN2A *locus in TC-32 and the high-level amplification of the *MDM2 *locus in OSA were estimated (Table 2). Copy number plots of the homozygous deletion of the *CDKN2A *locus as well as the heterozygous deletion of 9p21.3-p21.2 in TC-32 are shown in Additional file 2 for all microarray platforms.

The values were in general similar between the replicate hybridisations for all platforms and regions. Agilent arrays showed the highest dynamic range, from average log_2 _ratio of -4.78 to 4.72 for the homozygous deletion of the *CDKN2A *locus and the high-level amplification of the *MDM2 *locus, respectively, and gave almost expected log_2 _ratios for all regions, close to the theoretical values (Table 2). Affymetrix results were second best for log_2 _ratios for regions of increased copy number, 3 and 4 copies, but not so good on regions of decreased copy number, 0 and 1 copies. The Illumina results deviated most from the expected log_2 _ratios for 3 and 4 copies (theoretical value 0.58 and 1.0, respectively), giving on average log_2 _ratios of 0.22 and 0.36, respectively, while the Nimblegen data deviated most for 0 and 1 copies (theoretical value <-1 and -1.0, respectively), giving on average log_2 _ratios of -0.87 and -0.46, respectively. For the high-level amplification of the *MDM2 *locus in OSA, expected to have a log_2 _ratio well above 3 based on previous results [19,20], Nimblegen arrays gave the second best values, average log_2 _ratio of 2.52, whereas Affymetrix and Illumina arrays gave average log_2 _ratios of 1.93 and 1.24, respectively.

A regression line was calculated for the measured average log_2 _ratios of the regions representing 1, 2, 3 and 4 copies, and the slope and R^2 ^values are given in Figure 1. The regions representing 0 and > 10 copies were omitted from the regression line, since the expected log_2 _ratio for the homozygous deletion is not an exact number (< -3) and the exact log_2 _ratio for the high-level amplification is unknown (> 3). All platforms showed a high linearity of the measured log_2 _ratios, but the slope of the regression line varied. The slope of the measurements for the Agilent arrays was closest to the theoretical value, followed by Affymetrix and Illumina arrays, whereas the measurements from the Nimblegen arrays deviated most.

In a previous study using lower resolution oligonucleotide microarrays from Affymetrix (250 K), Agilent (185 K) and Illumina (317 K), as well as BAC arrays (32 K), for screening chronic lymphocytic leukaemia, similar results were observed [9]. Agilent 185 K arrays and 32 K BAC arrays showed the highest dynamic range, where the Agilent arrays showed the most correct response to loss of one copy and gain of one copy, whereas the BAC arrays showed the most correct response to homozygous deletion. A notable difference in the scale of log_2 _ratios has also previously been observed between Agilent 44 K arrays, Illumina 109 K arrays and ROMA/Nimblegen 82 K arrays for screening breast cancer, with higher signals for the Agilent arrays [6]. Affymetrix 100 K and 250 K arrays also showed higher mean log_2 _ratio of chromosome X than Nimblegen 385 K arrays in sex-mismatched hybridisations of patients with submicroscopic genomic copy number variations [11]. In a previous study on melanoma cell lines, Agilent 185 K arrays showed the highest signals for 4 copies (average log_2 _ratio 0.86) as well as the highest signal to noise ratio, whereas Affymetrix 500 K arrays showed intermediate values (average 0.55) and Nimblegen 1500 K arrays showed the lowest values (average 0.37) [8].

Concerning the variation in log_2 _ratios within a chromosome or chromosomal region, Affymetrix arrays showed the highest standard deviation for the regions representing 2, 3 and 4 copies, whereas the other platforms showed equal variation. Illumina and Agilent arrays showed the highest standard deviation for the regions representing 1 and 0 copies, respectively (0.57 and 1.55). Nimblegen arrays showed the lowest standard deviations for the regions of loss, but this is most likely due to compression of the log_2 _ratios since the Nimblegen data deviated most for 0 and 1 copies. For the high-level amplification of the *MDM2 *locus, Agilent showed the lowest and Nimblegen the highest standard deviations (0.23 and 0.99, respectively).

The baseline variation has also been determined in previous studies. For the screening of chronic lymphocytic leukaemia, Affymetrix 250 K arrays, Agilent 185 K arrays and Illumina 317 K arrays showed similar average log_2 _ratio and standard deviation of a region with normal copy number (chromosome 1) [9]. However, when assessing the baseline variation in form of autocorrelation of the whole genome, Agilent showed the lowest variation, followed by Affymetrix and Illumina. For the previous study of patients with submicroscopic genomic copy number variations, Nimblegen 185 K arrays showed a higher standard deviation of the log_2 _ratios of the whole genome (excluding regions harbouring the variations) than the Affymetrix 250 K and 100 K arrays [11].

**Figure 2:**
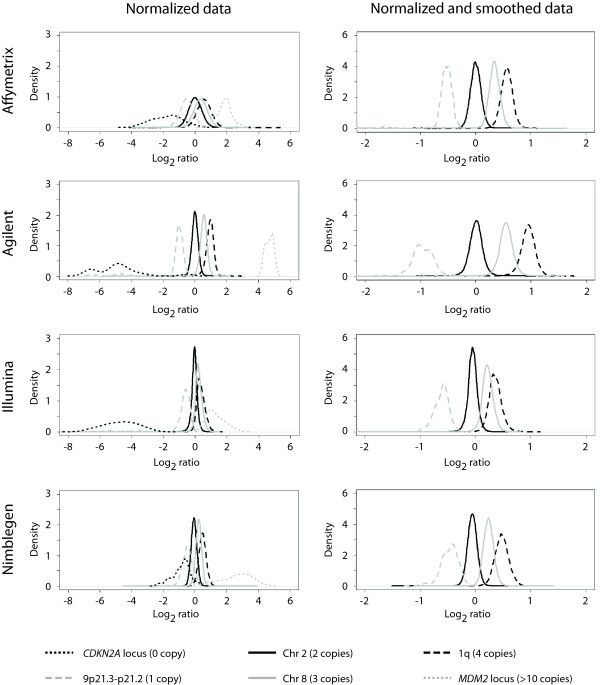
**Distribution of log_2 _ratios for specific chromosomes or chromosomal regions**. Distribution of log_2 _ratios of all probes within six specific chromosomes or chromosomal regions; the *CDKN2A *locus in 9p21.3, 9p21.3-p21.2, chromosome 2, chromosome 8, 1q (all in TC-32) and the *MDM2 *locus in 12q15 (in OSA) (representing 0, 1, 2, 3, 4 and >10 copies, respectively) for all microarray platforms for A) normalized data and B) normalized data that have been smoothed using Gaussian smoothing with a window size of 50 kb and standard deviation of 10 kb. Smoothing was not possible for the small regions representing 0 and >10 copies (the *CDKN2A *and *MDM2 *loci) due to insufficient number of probes.

The distribution of log_2 _ratios of all probes within the six specific chromosomes or chromosomal regions (representing 0, 1, 2, 3, 4 and >10 copies, respectively) for one hybridisation from all microarray platforms is shown in Figure 2. The replicate hybridisation showed a similar pattern (data not shown). The distribution is shown for both the normalized data and a smoothed version of the same data. The data were smoothed using Gaussian smoothing with a window size of 50 kb and standard deviation of 10 kb, in order to reduce the variation. Smoothing was not possible for the small regions representing 0 and >10 copies (the *CDKN2A *and *MDM2 *loci) due to insufficient number of probes. For the normalized data, the curves for the different copy number levels were by far best separated by the Agilent data, whereas the curves were highly overlapping for the Affymetrix data. However, smoothing of the data had a huge effect on the Affymetrix data, narrowing the distributions of the log_2 _ratios and thus better separating the curves. The smoothing also further improved the Agilent, Illumina and Nimblegen data, but to a smaller extent. The most difficult separation for all platforms was to distinguish between 3 and 4 copies, and this was particularly not easy with the Illumina data where the two curves were highly overlapping.

Similar results were observed in a previous study of melanoma cell lines, with the distribution of log_2 _ratios of regions of 2 and 4 copies [8]. The distribution was best separated for the Agilent 185 K and 44 K arrays, whereas the Affymetrix 500 K and 100 K arrays showed intermediate results. For the Nimblegen 1500 K arrays, the distribution of log_2 _ratios of regions of 2 and 4 copies was indistinguishable. However, in line with the observations in this study, smoothing of the data improved the results for all platforms, with a most profound effect for the Nimblegen 1500 K arrays and Affymetrix 100 K arrays, the two lowest ranking in terms of signal to noise ratios [8].

The size of the small aberrations of *CDKN2A *and *MDM2 *was estimated manually, and this revealed very similar results for all microarray platforms (Table 2). Only Affymetrix arrays showed a difference in size of the deletion of the *CDKN2A *locus between the replicate hybridisations for TC-32, detected to be 166 and 148 kb, respectively.

### Detection of DNA copy number aberrations

The aberrant regions examined for signal response to copy number were scored using the analysis software provided for each microarray platform with default settings. In addition, data from all platforms were exported into Nexus (BioDiscovery) in order to make an independent scoring of the aberrations. This software also has advantages when it comes to downstream integration with other genome-level data. In Nexus, all four platforms were analysed using the rank segmentation algorithm with default settings. Table 3 shows the scoring of the aberrant regions examined for signal response to copy number from the platform-specific analysis software as well as Nexus for one hybridisation from all platforms. The replicate hybridisation showed a similar pattern (data not shown). Detection of the homozygous deletion of the *CDKN2A *locus as well as the heterozygous deletion of 9p21.3-p21.2 in TC-32 using Nexus for all platforms is shown in Additional file 3.

**Table 3:** Detection of copy number of specific regions

Platform	0n: *CDKN2A* TC-32		1n: 9p21.3-p21.2 TC-32		3n: Chr 8 TC-32		4n: 1q TC-32		>10n: *MDM2 * OSA	
	
	Platform	Nexus	Platform	Nexus	Platform	Nexus	Platform	Nexus	Platform	Nexus
**Affymetrix Genome-Wide Human SNP array 6.0**	Yes	Yes	Yes	Yes	Yes	Yes	Yes	Yes	Yes	Yes

**Agilent Human Genome CGH 244A**	Yes	Yes	Yes	0n	Yes	Yes	Yes	Yes	Yes	Yes

**Illumina HumanExon510s-duo**	Yes	Yes	Yes	Yes	Yes	Yes	3n/4n	3n	Yes	Yes

**Nimblegen HG18 CGH 385 k WG Tiling v1.0^1^**	-	1n	-	Yes	-	Yes	-	3n	-	Yes

^1^Nimblegen's analysis software segments the data without scoring of copy number

Detection of copy number of five specific chromosomes or chromosomal regions representing 0, 1, 3 and 4 copies in TC-32, as well as high-level amplification in OSA, using the platform-specific software and the platform-independent software Nexus.

Using the platform-specific analysis software, Affymetrix, Agilent and Illumina scored the correct copy number level for all regions representing 0, 1, 2, 3 and 4 copies in TC-32, and indicated the high-level amplification in OSA. The only exception was the scoring of 4 copies of 1q using the Illumina data, where the software segmented the region into segments of mainly 3 copies and some smaller segments of 4 copies. For Nimblegen, the corresponding analysis software segments the data and displays them, without giving copy number scores.

Using the platform-independent Nexus software, all regions were determined to have the correct copy number for the Affymetrix data (Table 3). The region of 1 copy was over-scored as a homozygous deletion for Agilent, because of the low log_2 _ratios of this segment (average -0.97) and the threshold for homozygous deletion in Nexus (default log_2 _ratio < -1.0). The region of 4 copies was scored as one copy less for Illumina, most likely due to compression of the log_2 _values. For the Nimblegen data, Nexus detected the homozygous deletion as 1 copy and the 1q region as 3 copies instead of 4, most likely also due to compression of the log_2 _values.

In a previous study, Affymetrix 500 K arrays, Agilent 244 K arrays and Nimblegen 385 K arrays were compared for detection of submicroscopic constitutional aberrations [15]. In that study, using the corresponding analysis program, all 10 previously known abnormalities investigated were detected using the Agilent data, whereas one and three aberrations were not identified using the Affymetrix and Nimblegen data, respectively. However, using the software dChip in combination with an R script, all aberrations were detected using the Affymetrix data as well [15]. For the comparison of Affymetrix 500 K arrays, Agilent 44B arrays and Illumina Hap550 arrays, all known alterations in a leukaemia cell line were identified using both a platform-specific software and a platform-independent analysis (circular binary segmentation) [14].

The number of overall copy number aberrations in chromosome 1-22 detected by Nexus is given in Table 4, for both TC-32 and OSA for all microarray platforms and both replicate hybridisations. Detection of the copy number aberrations is given in Additional File 4. Nexus divides the copy number aberrations in four categories; homozygous copy loss, loss, gain and high copy gain depending on the log_2 _ratio of the segments. The number of detected copy number aberrations was in general similar between the replicate hybridisations, except for the categories gain and loss in TC-32 by Affymetrix, where the replicates varied with 31 and 36 aberrations, respectively.

**Table 4:** Detection of copy number aberrations

Platform	TC-32				OSA			
	
	Homozygous copy loss	Loss	Gain	High copy gain	Homozygous copy loss	Loss	Gain	High copy gain
**Affymetrix Genome-Wide Human SNP array 6.0**	19	45	40	21	18	100	101	72
	23	76	76	28	16	114	123	82

**Agilent Human Genome CGH 244A**	4	11	10	2	9	82	58	42
	4	8	10	2	13	103	65	37

**Illumina HumanExon510s-duo**	3	32	21	3	2	75	77	33
	3	33	19	2	4	86	78	36

**Nimblegen HG18 CGH 385 k WG Tiling v1.0**	0	7	15	2	1	31	62	36
	0	11	6	0	1	25	55	36

Number of copy number aberrations detected in chromosome 1-22 in TC-32 and OSA using the platform-independent software Nexus.

The number of detected aberrations varied between the platforms, in general showing that microarrays with a higher number of probes detect more segments of copy number aberrations. Affymetrix showed by far the highest number of aberrations, as expected with the 1.8 million probes on the array, and most additional aberrations compared to the other platforms were small regions (Table 4 and Additional File 4). However, Agilent, with only 236 k probes on the array, also showed a high number of small aberrations in the OSA cell line. Illumina identified approximately the same number of total aberrations as Agilent, whereas Nimblegen identified considerably less than the other platforms (Table 4). Some differences between the platforms were observed for larger regions, for instance the heterozygous deletion of 4q in OSA detected by Agilent, which was partly detected by Illumina and not at all by Nimblegen. Affymetrix detected several small regions within 4q as deletions (Additional File 4).

Similar results were observed in the analysis of the total number of chromosome segments altered in a leukaemia cell line, where the highest resolution arrays (Illumina Hap550) showed the highest number of identified segments, followed by Affymetrix 500 K arrays and Agilent 44B arrays [14]. On the other hand, for the screening of chronic lymphocytic leukaemia, the lowest resolution array (Agilent 185 K) detected the highest number of platform-specific copy number aberrations, followed by Affymetrix 250 K arrays and Illumina 317 K arrays [9]. Most of these aberrations were smaller segments. For the aberrations detected in common by two of the platforms, most often the Affymetrix and Agilent platforms showed concordant results [9]. A comparison of copy number aberrations detected in 18 melanoma cell lines by Affymetrix 500 K arrays and Agilent 244 K arrays showed a similar number of total aberrations detected with a 29% overlap between the two platforms [8].

### Scoring of loss of heterozygosity

An advantage of the Affymetrix and Illumina platforms is that they also provide global polymorphism information, and thus can indicate regions of LOH that could be involved in loss-of-function mutations, haploinsufficiency, etc. SNP analysis of the Affymetrix and Illumina data was performed in Nexus using the SNP-FASST segmentation algorithm with default settings.

In general, Affymetrix detected slightly more regions of allelic changes overall for both samples, but both platforms detected allelic changes in the regions with copy number aberrations scored by Nexus. In addition, regions of copy number-neutral allelic changes were identified, and detection of the copy number-neutral LOH of 1q in OSA using Nexus is shown in Additional file 5. Nexus divides the detection of allelic changes in two categories; LOH and allelic imbalance, depending on the distribution of the allelic ratio plot. For the copy-number neutral LOH of 1q in OSA, as well as other similar regions, the allelic changes were scored as LOH for the Illumina data, whereas the allelic changes were only scored as allelic imbalance for the Affymetrix data, due to a less defined distribution of the allelic ratio plot. The allelic ratios of the Illumina SNP data were overall better separated and thus more precisely scored, but all the allelic changes identified using the Illumina data were also identified using the Affymetrix data. Thus, the two platforms both perform well in detecting regions of allelic imbalance based on the SNP data.

Detection of LOH has previously been compared for Affymetrix 250 K arrays and Illumina 317 K arrays for chronic lymphocytic leukaemia [9]. Most loci were concordant between the two platforms, especially for regions > 4 Mb, but more differences were observed for smaller regions. The Illumina arrays showed in general a higher detection rate, in contrast with this study, but also a lower noise level in the LOH analysis, which was also observed in this study.

## Conclusions

All microarray platforms performed well in terms of reproducibility, delimiting small amplifications and deletions, as well as scoring the aberrations. Agilent microarrays showed better linearity and dynamic range since the measured log_2 _ratios using these platforms were closer to the expected ratios. The platform-specific analysis software identified in general correct copy numbers, whereas using a platform-independent analysis algorithm, correct copy numbers were determined mainly for Agilent and Affymetrix microarrays, implying more robust data. Bone tumours like osteosarcomas are heterogeneous tumours with complex karyotypes that may be difficult to interpret, and it is of importance to be able to well separate the copy number levels and detect copy number changes in subpopulations. Less complex tumours will also benefit from an increased linearity and dynamic range by allowing reliable detection of small subpopulations of cells with DNA copy number changes within relatively homogenous tumours. Taking all this into consideration, the Agilent and Affymetrix microarray platforms were found to be a better choice for mapping DNA copy numbers in bone tumours, the latter having the advantage of also providing heterozygosity information.

## Competing interests

The authors declare that they have no competing interests.

## Authors' contributions

SHK participated in the microarray experiments and analyses and drafted the manuscript, KS participated in the microarray analyses, conceived of the study, participated in its design and coordination, AHBP participated in the microarray experiments, HR participated in the microarray analyses, AMCJ conceived of the study, OM conceived of the study, participated in its design and coordination and helped to draft the manuscript, LAMZ carried out the microarray analyses, conceived of the study, participated in its design and coordination and helped to draft the manuscript. All authors read and approved the final manuscript.

## Supplementary Material

Additional file 1 **Scatter plot of log_2 _ratios and the correlation coefficient for the replicate hybridisations of TC-32 and OSA for all microarray platforms**. Click here for file

Additional file 2 **Copy number plot of the homozygous deletion of the *CDKN2A *locus and the heterozygous deletion of 9p21.3-p21.2 in TC-32 for all microarray platforms**. Click here for file

Additional file 3 **Detection of the homozygous deletion of the *CDKN2A *locus and the heterozygous deletion of 9p21.3-p21.2 in TC-32 using Nexus for all microarray platforms**. Click here for file

Additional file 4 **Detection of copy number aberrations in chromosome 1-22 in TC-32 and OSA using Nexus for all microarray platforms**. Click here for file

Additional file 5 **Detection of the copy number-neutral LOH of 1q in OSA using Nexus for the Affymetrix and Illumina platforms**. Click here for file
