# Supplementary figures and images for: Evaluation of high-resolution microarray platforms for genomic profiling of bone tumours
Source: BMC Res Notes. 2010 Aug 8;3:223. doi: 10.1186/1756-0500-3-223 (PMC2929238; doi:10.1186/1756-0500-3-223)

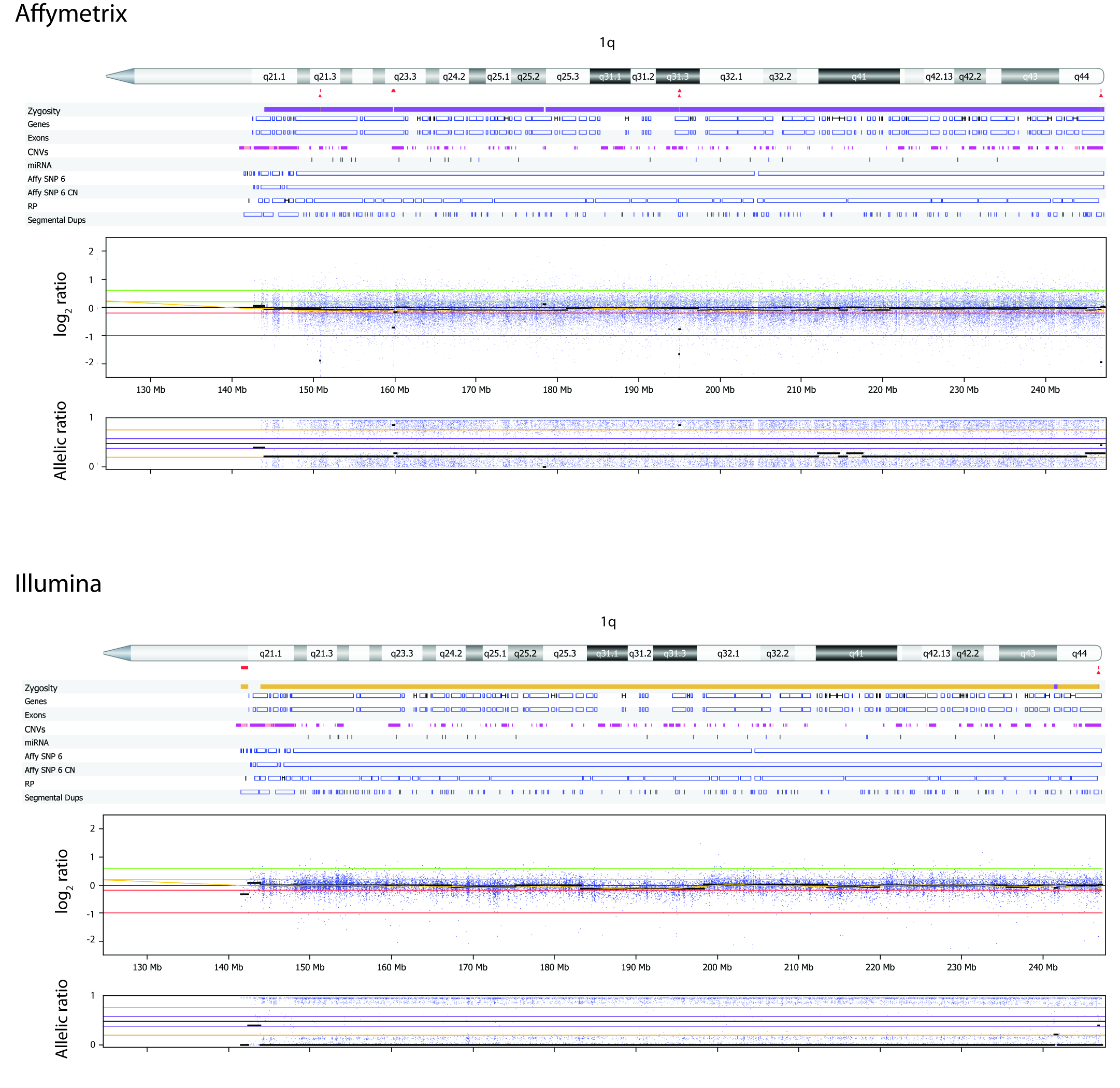

Supplement: Additional file 5 — Detection of the copy number-neutral LOH of 1q in OSA using Nexus for the Affymetrix and Illumina platforms. [file 1756-0500-3-223-S5.TIFF]
